# Bodies under stress—a psychological parallel mediation model between daily LGBTQ + heterosexism and eating disorder risk

**DOI:** 10.1007/s40519-026-01817-9

**Published:** 2026-01-16

**Authors:** Fabrizio Santoniccolo, Maria Noemi Paradiso, Tommaso Trombetta, Luca Rollè

**Affiliations:** 1 Department of Psychology, University of Turin, Turin, Italy; 2 School of Health, Medicine and Life Sciences, University of Hertfordshire, Hatfield, UK; 3 Institute of Applied Psychology, University of Social Science, Łódź, Poland

**Keywords:** Minority stress, Eating disorders, Body image, Emotion regulation, Self-esteem, Shame

## Abstract

**Purpose:**

LGBTQ + people have shown health disparities compared to heterosexual and cisgender people in eating disorders. How these disparities are determined, however, is an understudied area. Through the use of a psychological mediation framework, this study aims to explore how daily heterosexist experiences related to one’s LGBTQ + identity may determine eating disorder risk.

**Methods:**

376 LGBTQ + people from Italy responded to self-report questionnaires regarding daily heterosexist experiences, eating behaviors and associated factors in an online anonymous survey. Descriptive, bivariate and mediation analyses were conducted using the “PROCESS” macro, including distress scores for heterosexist experiences, emotion dysregulation, self-esteem, shame, and eating disorder risk, controlling for body-mass index, age and socioeconomic status.

**Results:**

Statistically significant positive associations were found between distress related to heterosexist experiences, emotion dysregulation, shame and eating disorder risk. Mediation analyses found that the direct effect of heterosexist experiences on eating disorder risk was nonsignificant. The indirect effects of heterosexist experiences on eating disorder risk through emotion dysregulation (*B* = 0.041, *β* = 0.304, BootSE = 0.017, 95% CI [0.006, 0.078]) and low self-esteem (*B* = 0.092, *β* = 0.089, BootSE = 0.023, 95% CI [0.049, 0.145]) were significant. The indirect effect through shame was nonsignificant.

**Conclusions:**

Heterosexist experiences seem to have significant indirect effects on eating disorder risk through emotion dysregulation and low self-esteem. Policies for reducing harassment, discrimination and violence related to sexual orientation and gender identity in institutional, organizational and social contexts may help prevent negative health outcomes in LGBT + people. Clinical contexts may benefit from considering the effects of minority stress.

**Level of evidence:**

Level 3–Observational cross-sectional study.

## Introduction

### The mental health burden of heterosexist experiences

Heterosexist experiences can be defined as those negative experiences deriving from the fact that one’s LGBTQ + 1identity deviates from the heterosexual and cisgender identity matrix that is socially considered the default. These are derived from the general phenomenon of heterosexism, that is, prejudice against any form of non-heterosexual behavior, relationship or community [1] as well as non-cisgender identities and gender presentations [3]. A number of phenomena can be categorized under heterosexism, such as discrimination, bias-motivated attacks and violence, familial strife regarding one’s relationship or identity and many more [4]. These are an unfortunately common occurrence in daily life for LGBTQ + people on an international level [5]. The Italian context in particular presents structural factors acting as minority stressors, such as the absence of LGBTQ + -specific protections and civil rights [6], as well as a sociopolitical climate with frequent bias-motivated attacks, discrimination and negativity towards LGBTQ + people [7].

Across the last two decades, a number of studies have highlighted disparities in the health levels of LGBTQ + people [8, 9]. Minority stress [10, 11]–that is, the presence of additional stress factors specific to LGBTQ + people–has been hypothesized to be a form of heterosexism that is a key determinant of this health disparity, a hypothesis that has received substantial empiric support for a variety of mental health phenomena [12].

### The impact of minority stress on disordered eating

Several studies have been published that give empirical support to application of minority stress theory for disordered eating and eating disorders as well. In both Sexual and Gender Minorities (SGMs), associations were found between forms of minority stress (e.g., discrimination, victimization, enacted stigma) and several forms of disordered eating [13] as well as with body image concerns [14], a known risk factor for eating disorders. In particular, eating disorder risk in LGBTQ + people has been found to be roughly twice that of heterosexual/cisgender people, and to be higher in people who had experiences of discrimination [15].

Furthermore, some studies have attempted to understand how these associations by surveying their impact on aspects such as social, coping, interpersonal and cognitive psychological processes. Minority stress theory has been extended through a Psychological Mediation Framework (PMF) [16] that attempts to take into account how in addition to their direct effects minority stressors can impact the person’s intrapsychic processes indirectly, explaining the disparities in prevalence found in LGBTQ + people. The general idea of the PMF is that the effects of stigma related to one’s LGBTQ + identity (e.g., through repeated exposure to heterosexist experiences in daily life) can negatively influence some of the individuals’ general psychological processes that are, in turn, linked to risks in mental health. Differently from how it is treated in Minority Stress Theory, stress in the PMF is theorized to be an inherently sufficient stand-alone predictor rather than a mediator, drawing inspiration from empirical results in general stress literature [17, 18]. For example, exposure to the stigma linked to LGBTQ + identity may deplete one's cognitive resources for regulating one’s emotions [19]. In turn, this may heighten risks related to mental health such as depression [20]. How stress affects coping and emotion regulation, social and interpersonal processes as well as cognitive processes may explain how stigma gets “under the skin” of LGBTQ + people.

In the case of disordered eating, several indirect effects have been previously surveyed such as body surveillance, body shame and negative affect [13]. However, aspects of this process have remained underexplored in minority stress literature. Several key risk factors in determining and maintaining eating disorders have been identified over the decades, which include, among many, low self-esteem, high levels of shame and disrupted emotion regulation processes [21]. These possible mediators are coherent with the PMF’s hypotheses around processes of stress generation and how they may heighten mental health risk. Exploring their possible mediating role could prove useful to managing eating disorder risk and reduce health disparities.

### Aims

The study aims to examine the link between heterosexist experiences and eating disorder risk while surveying the possible mediating role of emotion regulation, self-esteem and shame, informed by the PMF [16].

To the best of the authors’ knowledge, while some studies exist on the relationship between minority stress and disordered eating [13], no studies have been carried out yet on daily heterosexist experiences and eating disorder risk. These specific mediators were chosen through a combination of coherence with the PMF (i.e., emotion regulation processes, cognitive processes), coherence with available eating disorder literature (i.e., which factors are known to be associated with eating disorder risk) and gaps in the available literature (i.e., which factors have not already been studied through the PMF). Given its structural stigmatization of LGBTQ + identities, the Italian context warrants special attention in this regard as well, and to the authors’ knowledge no studies of this kind have been conducted in the Italian context.

### Hypotheses

- H_1_: Heterosexist experiences are positively and directly associated with eating disorder risk.
- H_2_: Heterosexist experiences are positively and directly associated with feelings of shame, low self-esteem and emotion dysregulation
- H_3_: Feelings of shame are positively and directly associated with eating disorder risk
- H_4_: Low self-esteem is positively and directly associated with eating disorder risk
- H_5_: Emotion dysregulation is positively and directly associated with eating disorder risk
- H_6_: Emotion dysregulation mediates the relationship between heterosexist experiences and eating disorder risk
- H_7_: Low self-esteem mediates the relationship between heterosexist experiences and eating disorder risk
- H_8_: Shame mediates the relationship between heterosexist experiences and eating disorder risk

**Fig. 1:**
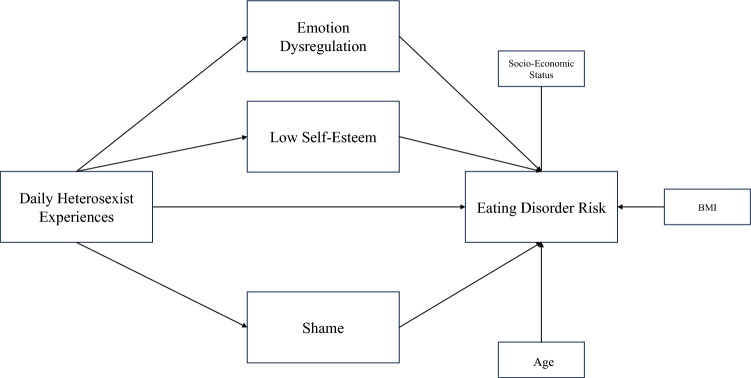
Hypothesized parallel mediation model

The hypothesized parallel mediation model is synthesized in Fig. 1 (including covariates). Covariates were selected for their influence according to existing literature mediators and eating disorder risk. We expected a higher BMI, a lower socioeconomic status and a lower age to be associated with higher levels of all main variables. Relationships between covariates and mediators have been omitted for simplicity.

## Methodology

### Participants

The initial sample was composed of 386 participants in the “Factors associated with the eating behaviours of LGBTQ + people” project carried out in Italy between June 2024 and April 2025.

Three inclusion criteria were defined:

- Identifying as any LGBTQ + identity regarding sexual orientation or gender identity (e.g., gay, lesbian, bisexual, pansexual, queer, trans/transgender, non-binary, gender non-conforming, genderfluid, etc.)
- Being at least 18 years old
- Being able to read, understand and write in the Italian language
  One exclusion criterion was defined:
- Identifying as heterosexual while declaring being cisgender (i.e., having a gender identity that matches one’s assigned gender at birth)

### Procedure

The study procedures were carried out in accordance with the ethical standards of the American Psychological Association and the 1964 Declaration of Helsinki. The study was approved by the Bioethical Committee of the University of Turin on 30/04/2024 with protocol number 0307736. An online survey was developed using the LimeSurvey platform running version 6.3.8 + 231,204. The link to the survey was disseminated through public flyers at relevant places and events (e.g., LGBTQ + friendly establishments, local Pride events), the researchers’ personal and professional networks, emails, and through collaboration with gyms and LGBTQ + associations. The study also employed snowball sampling by asking the participants to send the link and/or flyer to other LGBTQ + people they may know, disseminating the study through word of mouth. Participants were informed of the general topic of the study, along with possible risks and benefits connected to participation, asked to confirm being at least 18 years old and asked for consent to participation. Participation was voluntary and completely anonymous. No reward or incentive was offered for participation in the study.

Participants were asked to fill out a sociodemographic sheet, in which they were asked which word best described their sexual orientation and gender identity, as well as to confirm whether they were a trans or otherwise gender diverse person. Participants who reported both a heterosexual and cisgender identity were not allowed to participate in the study.

Participants responded to a battery of questionnaires surveying aspects related to experiences as LGBTQ + people, body image and eating behaviours. Whenever possible, validated questionnaires in the Italian language were used. The full questionnaire took participants an average of 30 min to complete.

A number of response quality control measures were employed. Two attention checks were added at random points throughout the survey (e.g., “if you are still reading attentively, select ‘Often’ as the answer”). Participants who failed one or more attention checks were excluded from the analyses (6 people removed). The participants’ response time was also screened: responses that had significantly quicker response time compared to the mean using the inter-quartile rule for mild outliers (more than 1.5 standard deviations faster than the mean) were checked for response sets (0 people removed) and incoherent responses (2 people removed). Participants also developed an anonymous participant code, which was used to screen for duplicate answers. This participation code was developed according to suggested guidelines from Audette and colleagues [22] and included 5 bits of information: birth month, initial of one’s oldest parent’s name, initial of one’s youngest parent’s name, second letter of the participant’s name, number of siblings born before the participant. Finally, participants who entered invalid or insufficient information in the “sexual orientation” or “gender identity” (e.g., using the “Other” field to enter an answer that is not relevant to the respective text field) were excluded (2 people removed). A total of 10 people were removed from the dataset. After this data quality screening, 376 participants were retained in the current analyses.

### Measures

*Eating disorder inventory–3* (EDI-3) [23]: participants responded to 91 items evaluating eating behaviours, attitudes, body image and other psychological aspects connected to eating disorders. It has been validated in the Italian language [24] and has been found to have very good psychometric properties [21, 25]. The EDI-3 uses a Likert 5-point scale assessing the participant’s perceived frequency of the relevant items from “never” to “always”. In this study, three subscales were used.

The Low Self-Esteem subscale involves items related to a low evaluation of one’s subjective personal worth (such as «I feel inadequate» or «I feel secure about myself», reverse scored). Higher scores suggest lower self-esteem.

The Emotion Dysregulation subscale involves items related to one’s ability in recognizing and regulating one’s emotions (such as «Other people would say that I am emotionally unstable» or «I don't know what's going on inside me»). Higher scores suggest lower abilities in emotion regulation.

The Eating Disorder Risk Composite (EDRC) scale score is the sum of the scores from three additional subscales from the EDI-3: the Bulimia subscale, which measures behaviours and attitudes related to overeating (such as «I stuff myself with food»); the “Drive for Thinness” subscale which measures one’s desire to attain a thinner body (such as «I am preoccupied with the desire to be thinner»); the Body Dissatisfaction subscale which measures one’s current lack of satisfaction with body parts and their body as a whole (such as «I feel satisfied with the shape of my body», reverse scored). Higher total scores suggest higher general eating disorder risk.

*Daily Heterosexist Experiences Questionnaire* (DHEQ) [4]: participants responded to 38 items related to daily heterosexist experiences relevant to being LGBTQ + specifically (some scales from the original were omitted). The scale has been previously validated in the Spanish [5] and Polish [26] languages, showing adequate psychometric properties. The scale allows participants to report on whether they had a specific experience or not. Example items include «Being verbally harassed by strangers because you are LGBT + » (Harassment and discrimination subscale), «Being rejected by your mother for being LGBT + » (Family of origin subscale), and «Hiding part of your life from other people» (Vigilance subscale). The scale was scored in the “Distress” mode, as participants reported on their perceived distress level for each experience (depending on the level of distress, ranging from “did not happen/does not apply to me” and “not at all”–both scored 1 point–to “extreme”–5 points). The scale was translated into Italian: two authors (FS and TT) prepared the translation independently and compared results, discussing and resolving any discrepancies with a third author (LR) until unanimous consent was achieved. For this study, the total score was computed, adding up the points from the subscales administered in this questionnaire (Vigilance, harassment and discrimination, gender expression, victimization, family of origin, vicarious trauma, isolation). Higher total scores suggest higher distress related to LGBTQ + -related heterosexist experiences.

*Personal Feelings Questionnaire–2* (PFQ-2) [27]: the Italian validation of the PFQ-2 [28] was administered. The PFQ-2 has been used with LGBTQ + people before [29] and has been found to have good psychometric properties [27, 30]. The PFQ-2 is comprised of 22 items measuring a person’s tendency to experience feelings of guilt and shame (shame-proneness and guilt-proneness) by asking them to rate how frequently they experience each sensation (e.g., «Feeling humiliated» or «Feeling you deserve criticism for what you did») on a 5-point Likert-type scale ranging from “Never” to “All the time or almost always”. Raw scores were computed according to instructions for the Shame subscale. Higher scores are representative of more common feelings of shame.

*Body-Mass Index* (BMI): using participants’ self-reported height and weight, their BMI (ratio between one’s mass and squared height) was computed.

### Data analysis

All data analyses were performed in RStudio using R version 4.5.1 [31]. Two-tailed bivariate correlations between the variables were tested using Pearson’s correlation (r), and results were interpreted according to original guidelines. The PROCESS macro version 5.0 for R [32] was used for mediation analysis, employing Model 4. The total DHEQ score was entered as the independent variable. The Low Self-Esteem, Emotion Dysregulation and Shame scores were entered as parallel mediators. The EDRC score was entered as a dependent variable. Age (continuous), BMI (continuous), and socio-economic status (ordinal) were entered as covariates, in accordance with the literature highlighting their influence on sexual identity, body image and eating behaviours.

Data were checked for consistency with assumptions of linear regression through visual checking of P-P and Q-Q plots and through checking of Variance Inflation Factor (VIF). No significant multicollinearity was found, as VIF values of predictor variables was less than 5 [33]. Specifically, VIF values were as follows: Emotion Dysregulation (1.50), Shame (2.34), Low Self-Esteem (2.20), DHEQ total score (1.19), age (1.06), socio-economic status (1.03), BMI (1.05). Due to the violation of the multi-normality assumption, a heteroskedasticity-consistent estimator was selected in PROCESS (HC3; [34, 35]). Bootstrap estimation with 5000 samples was used to determine 95% Confidence Intervals (CIs); effects were considered significant when CIs did not include 0.

#### Subscale reliability

To estimate the reliability of the employed subscales, McDonald’s *ω* [36] was computed using the OMEGA macro [37] through forced single factor maximum likelihood analysis. Computed values are available in Table 1.

**Table 1:** McDonald’s *ω* values for employed subscales

Subscale	McDonald’s *ω*
Vigilance¹	0.835
Harassment and discrimination¹	0.801
Gender Expression¹	0.730
Victimization¹	0.597
Family of Origin¹	0.780
Vicarious Trauma¹	0.787
Isolation¹	0.636
Bulimia²	0.893
Drive for Thinness²	0.929
Body Dissatisfaction²	0.885
Low Self-Esteem	0.911
Emotion Dysregulation	0.814
Shame	0.892

¹: Translated in Italian. Used in computing DHEQ total score

²: used in computing EDRC

Most employed subscales showed sufficient, good or excellent reliability save for the Victimization subscale.

#### Missing values

5 values were missing regarding socio-economic status, 1 value was missing regarding age, and 1 value was missing regarding education status. Missing values were replaced with the sample’s median values due to the small quantity of missing data.

#### Group differences

Group differences for gender identity across the main study variables were checked through independent samples Mann–Whitney U tests.

## Results

### Descriptive analyses

#### Participant information

The final sample selected for data analysis is comprised of 376 LGBTQ + people. The sociodemographic characteristics are described in Table 2.

**Table 2:** Sociodemographic characteristics of the participants

Gender identity	*N*	%
Woman	190	50.5
Man	118	31.4
Non-binary	36	9.6
Gender non-conforming	15	4
Genderfluid	11	2.9
Other gender identity	6	1.6
Sexual Orientation	*N*	%
Bisexual	113	30.1
Gay	88	23.4
Lesbian	74	19.7
Pansexual	53	14.1
Asexual	24	6.4
Queer	13	3.5
Heterosexual^a^	7	1.9
Other sexual orientation	4	1.1
Socio-Economic Status^1^	*N*	%
Insufficient	7	1.9
Precarious	56	14.9
Sufficient	187	49.7
Comfortable	113	30.1
More than well-off	8	2.1
Education Status^2^	*N*	%
No formal education	0	0
Primary education	0	0
Lower secondary education/Middle school	3	0.8
Upper secondary education / Diploma	191	50.8
Bachelor’s degree/First-level University degree	104	27.7
Master’s degree/Second-level University degree	60	16
Doctorate/Specialization/Third-level University degree/Postgraduate studies	17	4.5
Relationship status	*N*	%
Single	174	46.3
Couple	113	30.1
Cohabiting couple	58	15.4
Married/Civil union	7	1.9
Polyamorous relationships/One or more non-monogamous relationships	18	4.8
Other relationship status	5	1.3

¹: 5 missing responses. Percentages of *N* = 371

²: 1 missing response. Percentages of *N* = 375

^a^: people who reported diversities in their gender identity (e.g., being trans, non-binary, etc.)

61 participants (16.2%) additionally declared being trans. Furthermore, physical self-reported characteristics of the participants are described in Table 3.

**Table 3:** Physical characteristics of the participants

Variable	Range	Mean	SD
Age (in years)¹	18–57	25.82	6.29
Height (in centimetres)	152–194	169.31	8.61
Weight (in kilograms)	30–160	67.14	17.04
Body-Mass Index	12–54	23.29	5.07

¹: One missing response. SD: Standard Deviation

#### Study variables

Descriptive statistics about key study variables are described in Table 4.

**Table 4:** Measured range, means and standard deviations of the main variables

Variable	Range	Mean	SD
DHEQ Total Score	42–165	81.3	20.8
Low Self-Esteem	0–24	10.1	6.32
Emotion Dysregulation	0–29	6.48	5.82
Shame	0–40	18.1	8.26
Eating Disorder Risk Composite	0–95	32.9	21.6

Values are expressed in non-standardized raw scores. SD: Standard Deviation

#### Group differences

The sample was checked for statistically significant differences across cisgender vs Trans and Gender Diverse (TGD) identities. TGD people (Mean Rank = 224.66) reported a significantly higher level of distress when compared to cisgender people (Mean Rank = 177.29) related to heterosexist experiences (*U* = 15,990; *p* < 0.001). The same was found for low self-esteem (*U* = 150,008; *p* < 0.05): TGD people showed significantly lower self-esteem (Mean Rank = 213,63) when compared to cisgender people (Mean Rank = 180,71; please note: higher mean ranks indicate lower self-esteem as per the scoring method). Similarly, TGD people (Mean Rank = 210.97) were found to report significantly higher shame (*U* = 14,771; *p* < 0.05) when compared to cisgender people (Mean Rank = 181.53). Tests for group differences in emotion dysregulation and eating disorder risk were not statistically significant (*p* > 0.05).

### Correlational analyses

Table 5 shows the correlations between the main study variables at the bivariate level.

**Table 5:** Two-tailed Pearson’s Correlations between main variables

Variable	DHEQ Total Score	Low Self-Esteem	Emotion Dysregulation	Shame	Eating Disorder Risk Composite
DHEQ Total Score	–				
Low Self-Esteem	0.264***	–			
Emotion Dysregulation	0.316***	0.501***	–		
Shame	0.341***	0.722***	0.523***	–	
Eating Disorder Risk Composite	0.168**	0.510***	0.374***	0.429***	–

^*^: *p* < 0.05; **: *p* < 0.01; ***: *p* < 0.001

Results were consistent with H_1_ through H_5_ at the bivariate level. All main variables were positively and significantly correlated with each other, ranging from small to large correlations. A large correlation was found between Shame and Low Self-Esteem.

### Mediation analyses

The main linear regression model (all predictors and covariates included) was statistically significant and explained 41% of the variance in the EDRC score (R2: 0.410, *F* = 28, *p* < 0.0000).

#### Main variables in the regression models

In contrast with H_1_, heterosexist experiences did not have a significant association with eating disorder risk (95% CI [− 0.037, 154]) in the regression model. However, in accordance with H_2_, heterosexist experiences had a significant positive association with emotion dysregulation (*B* = 0.085, *β* = 0.304, SE = 0.015, 95% CI [0.055, 0.115]), low self-esteem (*B* = 0.079, *β* = 0.259, SE = 0.015, 95% CI [0.049, 0.108]) and shame (*B* = 0.131,, *β* = 0.331, SE = 0.022, 95% CI [0.087, 0.176]). In contrast with H_3_, shame did not have a significant relationship with eating disorder risk (95% CI [− 0.077, 0.564]). In accordance with H_4_ and H_5_, emotion dysregulation (*B* = 0.481, *β* = 0.129, SE = 0.193, 95% CI [0.081, 0.880]) and low self-esteem (*B* = 1.173, *β* = 0.344, SE = 0.193, 95% CI [0.794, 1.553]) had significant positive associations with eating disorder risk.

#### Total, direct and indirect effects

The total effect of the mediation model was found to be significant (*B* = 0.224, *β* = 0.159, SE = 0.050, 95% CI [0.125, 0.323], *p* < 0.0000).

Regarding covariates in the total effect model, BMI (*B* = 1.747, *β* = 0.411, SE = 0.251, 95% CI [1.252, 2.242) was positively correlated with eating disorder risk, while age was negatively correlated (*B* = − 0.382, *β* = − 0.111, SE = 0.154, 95% CI [− 0.686, − 0.079]). Socioeconomic status (95% CI [− 4.011, 1.368]) was nonsignificant.

In contrast with H_1_, no direct effect of heterosexist experiences was found on eating disorder risk (95% CI [− 0.037, 0.154]).

In accordance with H_2_, H_4,_ H_5_, H_6_ and H_7_, positive indirect effects between heterosexist experiences and eating disorder risk were found through low self-esteem (*B* = 0.092, *β* = 0.089, BootSE = 0.024, 95% CI [0.049, 0.145]) and emotion dysregulation (*B* = 0.041, *β* = 0.039, BootSE = 0.017, 95% CI [0.006, 0.078]). In contrast with H_8_, the indirect effect through shame was not significant (95% CI [− 0.009, 0.081]).

**Fig. 2:**
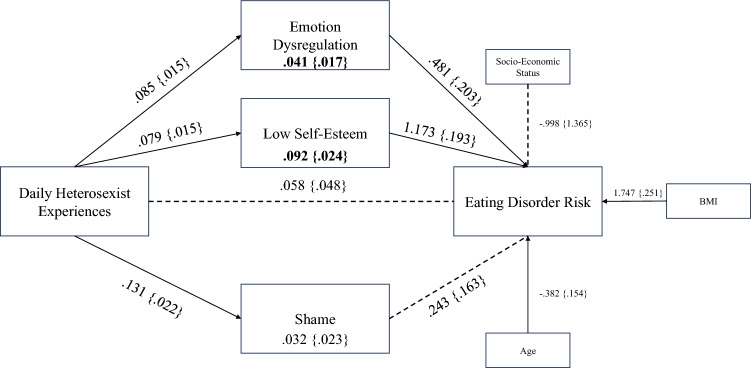
Measured parallel mediation model Bold: statistically significant indirect effect coefficient. Dotted line: 95% CI included 0–relationship not statistically significant. Figure uses unstandardized coefficients (B) for all relationships. Standard Errors (for regression coefficients) and Bootstrap Standard Errors (for indirect effects) included within curly brackets

Figure 2 synthesizes the measured mediation model in a graphical representation.

## Discussion

The vast majority of the study’s hypotheses were verified.

In contrast with the initial hypothesis, the direct effect of heterosexist experiences was not significant. Instead, according to most of the other hypotheses, the measured parallel mediation model showed mediation through the indirect effects of low self-esteem and emotion dysregulation. Consistently with the PMF [16], indirect effects were found through intrapsychic characteristics that are associated with higher eating disorder risk with small but significant coefficients for emotion dysregulation and low self-esteem. The indirect effect through shame was not verified–while heterosexist experiences do seem to increase shame, the mediation analysis did not find an indirect effect of this relationship on eating disorder risk. To summarize, the study results seem to imply that heterosexist experiences are associated with eating disorder risk through their effects on emotion dysregulation and low self-esteem, but not through their effects on general shame-proneness.

Heterosexist experiences were relatively commonplace in our sample, and distress related to them was significant (mean score of 81.3 out of a maximum of 165). The detected minimum range (42) also paints heterosexist experiences as universal in our sample, as all participants reported having experienced at least one of them. This is consistent with recent international trends: despite recent progress in acceptance of some aspects of LGBTQ + identities through western society [38, 39], discrimination, harassment, violence and bias-motivated attacks remain frequent [40]. Along with the erosion of civil rights and frequent targeting by major political actors [41], heterosexism remains a powerful force hindering the mental health of LGBTQ + people. Data from this study confirms this affects eating disorder risk as well, which is especially worrying when considering its mortality risk and healthcare burden [42].

Self-esteem levels seem to be affected by heterosexist experiences and to be associated with heightened eating disorder risk in turn. The fact that an important part of one’s identity is perceived to be rejected and stigmatized by society as well as important people in one’s life (e.g., as with family-related heterosexist experiences) may play an important role in lowering self-esteem, which has been found to be a significant risk factor in eating disorders [43]. This result confirms the role of negative self-schemas (cognitive components of the PMF) as important mediators in LGBTQ + people’s mental health, for eating disorder risk in particular.

The importance of emotion regulation is known in eating disorders, painting difficulties in emotion regulation as a risk factor [44]. Additionally, individuals’ capacity for emotion regulation could be conceived of as a potentially finite resource. Having to frequently cope with negative affect due to heterosexist experiences could increase eating disorder risk by depleting coping resources earlier [19]. This result as well confirms the role of emotion regulation processes as described in the PMF as key determinants of mental health risk.

The fact that shame was not significant in this model could be attributed to high levels of shared variance with low self-esteem despite not finding significant multicollinearity, as previous evidence paint it to be an important factor in the symptomatology of eating disorders [45]. The overlap in negative self-evaluation between low self-esteem and shame could have turned off the effect of shame towards eating disorder risk in this particular model. An additional interpretation could be that, when accounting for the heightening of negative self-evaluation through other variables (i.e., low self-esteem), only specific experiences of shame, such as body-related and food-related shame, would produce an effect on eating disorder risk rather than general shame-proneness as explored in this study. This would be coherent with previous meta-analytic analyses in which these particular forms were most commonly associated with eating disorder symptoms [45], as well as previous studies on body shame in LGBTQ + people [46]. Additionally, it could be possible that the role of general shame has differing importance in the various identities that make up our sample—for example, it could be that it is far more important in people identifying as women than for those identifying as men, and the presence of all identities in the sample render it non-significant.

Consistently with previous evidence highlighting mental health disparities for TGD people [47], the analysis of group differences highlighted significantly higher shame and lower self-esteem in TGD participants when compared to cisgender participants. Coherently with the PMF and the study results, it’s possible that the higher levels of shame and lower levels of self-esteem are connected to the experience of additional distress related to heterosexist experiences, which is indeed significantly higher in TGD participants.

### Policy implications and clinical recommendations

The results of this study confirm previous data regarding the mental health burden of heterosexist experiences for eating disorder risk and further recontextualize heterosexism as a public health issue. Structural protections and policies should be promoted at the local, national and international level aimed at preventing discrimination, violence and stigma related to sexual orientation and gender identity, which may prevent minority stress from affecting the health of LGBTQ + people. Promoting knowledge about LGBTQ + identities may reduce negativity and help foster contexts that are respectful of diversities in sexual orientation and gender identity, protecting individuals’ health.

In clinical work, focusing on minority stress has been previously suggested to be a promising endeavour [12, 48]. Treatments for disordered eating that are informed by minority stress theories have been developed with promising results [49]. Programs promoting knowledge and information around LGBTQ + identities may also prove useful in reducing the spread of negativity and promote contexts that protect individuals’ health through being respectful of diversities in sexual orientation. In this particular case, focusing on how heterosexist experiences may have affected a person’s self-esteem and strengthening emotion regulation processes may prove to be useful.

### Strength and limits

The study uses a strong theoretical framework with very good empirical support in the literature. Furthermore, several potential sources of bias in the dataset were addressed, such as inattentive responses, incoherent responses and response sets, as well as the possible confounding effects of sociodemographic variables. The employed measures were overall reliable. The use of bootstrap confidence intervals in data analysis should additionally strengthen the reliability of reported findings.

However, the study presents some important limitations. The study uses self-report measures, which have known limits. The main ones involve the possibility of distorted responses. For example, researcher acquiescence may be involved–that is, a person may respond in a way that tries to go along with the perceived hypotheses of the study [50]. Social desirability bias could also play a role—responding in a way that a person overly focused on pleasing others feels would be socially appropriate and desirable [51]. Respondent inattention may be a problem in this type of questionnaire [52], and despite our attempt to reduce the issue, within-questionnaire inattention may still have affected the results. BMI computation in this study could also have suffered from distortions in self-reporting of height and weight, as people tend to overreport on height and underreport on weight by a small margin [53]—this issue may bias the precision of the study’s BMI measurement. BMI also has its own limitations as a measure (e.g., lack of ability to differentiate between body fat and muscle).

The sample was homogeneous in several sociodemographic characteristics, showing a sufficient or better socioeconomic status, a relatively young mean age, and generally high levels of education. Additionally, the study involves many kinds of different LGBTQ + identities, which are known to have differences and specificities, as highlighted for gender identity in our sample by the U Mann–Whitney tests. Generalizability of results should be interpreted with caution.

While the causal order of the variables was theoretically defined and appears logically sound, the cross-sectional nature of the study makes it ill-fitted to provide certainty in the order of causal relationships. Specifically, given that the study does not evaluate how the relationships between variables evolve over time, it may be possible that the order of causal influence between variables would not verify this model’s exact relationships in a longitudinal study. The status of minority stress as predictor is unlikely to be in doubt, given the extensive literature for general stress as a sufficient predictor for heightened mental health risk [17, 18] and for minority stress specifically [13]. Furthermore, a previous longitudinal study has confirmed the predictive role of minority stress in eating pathology across different variables [54], giving additional credibility to these findings. The role of the chosen mediators as predictors of eating disorder risk, however, is less certain. It may be possible, for example, that body dissatisfaction (part of the eating disorder risk subscale) is a predictor in this relationship rather than a mediator, and it leads to lower self-esteem rather than the other way around. These possibilities can only be verified through a future longitudinal study.

Finally, the study uses an author-translated scale (DHEQ) as a measure for minority stress that has yet to be validated in the Italian language. Additionally, while its measured reliability appears good in most subscales, some (i.e., “Victimization”) were suboptimal, although it’s unclear whether this is due to the translation or due to the relatively low detected levels of victimization. Ideally, conducting this type of study again with a higher sample size or with a different sampling method so as to possibly detect broader levels of victimization would be advisable.

#### Future directions

More research on SGMs and with more diverse samples would be necessary to confirm these relationships. Low socioeconomic status in particular may be an additional axis of intersectional analysis [55], and has previously been associated with higher chances of eating disorders in SGMs [56]. How multiple marginalizations on several axes impact eating behaviours in LGBTQ + people may be an important area to explore that warrants further attention.

While minority stress theories have amassed a substantial body of empiric evidence, additional studies would be best to confirm our findings. Longitudinal studies in particular could provide confirmation of the order of causal relationships, since an overwhelming part of scientific literature on the topic consists of cross-sectional studies [13]. In this particular case, an additional exploration of shame-proneness in LGBTQ + people and how it affects eating disorder risk throughout time could be worthwhile and help clear up its role. Qualitative studies as well are very scarce on this topic [13]–conducting more may provide in-depth insights on experiences of LGBTQ + people regarding eating disorders and how they may be related to minority stress.

## Conclusions

Heterosexist experiences can heighten eating disorder risk via indirect effects through emotion dysregulation and low self-esteem. Preventing heterosexism, lowering minority stress and managing its effects on emotion regulation and self-esteem may help prevent risks for physical and mental health in LGBTQ + people.

### What is already known on this subject?

Minority stress is consistently associated with disordered eating behaviors and body image concerns.

### What does this study add?

Daily heterosexist experiences can increase eating disorder risk through their indirect effects on emotion dysregulation and low self-esteem. Managing the effects of minority stress on a clinical and policy level, as well as reducing the prevalence of heterosexist experiences, could improve the physical and mental health of LGBTQ + people and reduce eating disorder risk.

## Data Availability

The datasets analysed during the current study are available from the corresponding author Maria Noemi Paradiso upon reasonable request.

## Footnotes

1 People who identify as lesbian, gay, bisexual, trans, queer or more. For a more comprehensive definition of terms related to sexual orientation and gender identity, refer to the APA Dictionary of Psychology [1] or the INSIGHT project’s Good Practices Guide glossary [2].

## References

[1] American psychological association (2024) APA dictionary of psychology. https://dictionary.apa.org/. (Accessed 29 Apr 2024)

[2] Carpinelli L, Molinari M, Dimitris M, et al (2023) Appendix: glossary of terms. In: good practices guide for LGBTI+ inclusive healthcare. Filippos Paganis, pp 172–176

[3] Knutson D, Goldbach C, Kler S (2021) Heterosexism. In: The SAGE encyclopedia of trans studies. SAGE Publications, Inc., 2455 Teller Road, Thousand Oaks California 91320

[4] Balsam KF, Beadnell B, Molina Y (2013) The daily heterosexist experiences questionnaire: measuring minority stress among lesbian, gay, bisexual, and transgender adults. Meas Eval Couns Dev 46:3–25. 10.1177/0748175612449743 24058262 10.1177/0748175612449743 PMC3777637

[5] Ronzón-Tirado R, Charak R, Cano-González I (2023) Daily heterosexist experiences in LGBTQ+ adults from Spain: measurement, prevalence, and clinical implications. Psychosoc Interv 32:1–10. 10.5093/pi2022a15 37361633 10.5093/pi2022a15 PMC10268542

[6] ILGA Europe (2024) Italy–rainbow map

[7] OECD (2019) Society at a Glance 2019: OECD social indicators. OECD

[8] Clark KA, Schwartzman JM, Bettis AH (2024) Sexual and gender minority stress and clinical symptom severity in psychiatrically hospitalized adolescents. Psychiatr Res 334:115838. 10.1016/j.psychres.2024.115838 10.1016/j.psychres.2024.115838 PMC11753029 38452497

[9] Wittgens C, Fischer MM, Buspavanich P et al (2022) Mental health in people with minority sexual orientations: a meta‐analysis of population‐based studies. Acta Psychiatr Scand 145:357–372. 10.1111/acps.13405 35090051 10.1111/acps.13405

[10] Hendricks ML, Testa RJ (2012) A conceptual framework for clinical work with transgender and gender nonconforming clients: an adaptation of the minority stress model. Prof Psychol Res Pract 43:460–467. 10.1037/a0029597

[11] Meyer IH (2003) Prejudice, social stress, and mental health in lesbian, gay, and bisexual populations: conceptual issues and research evidence. Psychol Bull 129:674–697. 10.1037/0033-2909.129.5.674 12956539 10.1037/0033-2909.129.5.674 PMC2072932

[12] Frost DM, Meyer IH (2023) Minority stress theory: application, critique, and continued relevance. Curr Opin Psychol. 10.1016/j.copsyc.2023.101579 37270877 10.1016/j.copsyc.2023.101579 PMC10712335

[13] Santoniccolo F, Rollè L (2024) The role of minority stress in disordered eating: a systematic review of the literature. Eat Weight Disord 29:41. 10.1007/s40519-024-01671-7 38850334 10.1007/s40519-024-01671-7 PMC11162380

[14] Santoniccolo F, Trombetta T, Paradiso MN, Rollè L (2025) The relationship between minority stress and body image—a systematic review of the literature. Sex Res Soc Policy. 10.1007/s13178-025-01156-x

[15] Kamody RC, Grilo CM, Udo T (2020) Disparities in DSM-5 defined eating disorders by sexual orientation among US adults. Int J Eat Disord 53:278–287. 10.1002/eat.23193 31670848 10.1002/eat.23193

[16] Hatzenbuehler ML (2009) How does sexual minority stigma “get under the skin”? A psychological mediation framework. Psychol Bull 135:707–730. 10.1037/a0016441 19702379 10.1037/a0016441 PMC2789474

[17] Dohrenwend BP (2000) The role of adversity and stress in psychopathology: some evidence and its implications for theory and research. J Health Soc Behav 41:1–19 10750319

[18] Monroe SM (2008) Modern approaches to conceptualizing and measuring human life stress. Annu Rev Clin Psychol 4:33–52. 10.1146/annurev.clinpsy.4.022007.141207 17716038 10.1146/annurev.clinpsy.4.022007.141207

[19] Inzlicht M, McKay L, Aronson J (2006) Stigma as ego depletion: how being the target of prejudice affects self-control. Psychol Sci 17:262–269. 10.1111/j.1467-9280.2006.01695.x 16507068 10.1111/j.1467-9280.2006.01695.x

[20] Hatzenbuehler ML, McLaughlin KA, Nolen-Hoeksema S (2008) Emotion regulation and internalizing symptoms in a longitudinal study of sexual minority and heterosexual adolescents. Child Psychol Psychiatry 49:1270–1278. 10.1111/j.1469-7610.2008.01924.x 10.1111/j.1469-7610.2008.01924.x PMC2881586 18564066

[21] Clausen L, Rosenvinge JH, Friborg O, Rokkedal K (2011) Validating the eating disorder inventory-3 (EDI-3): a comparison between 561 female eating disorders patients and 878 females from the general population. J Psychopathol Behav Assess 33:101–110. 10.1007/s10862-010-9207-4 21472023 10.1007/s10862-010-9207-4 PMC3044826

[22] Audette LM, Hammond MS, Rochester NK (2020) Methodological issues with coding participants in anonymous psychological longitudinal studies. Educ Psychol Meas 80:163–185. 10.1177/0013164419843576 31933497 10.1177/0013164419843576 PMC6943988

[23] Garner DM (2004) Eating disorder inventory-3: professional manual. psychological assessment resources, incorporated

[24] Giannini M, Pannocchia L, Dalle Grave R, Muratori F (2008) Adattamento italiano dell’EDI-3. Eating Disorder Inventory-3. Giunti O.S.

[25] Punzi C, Tieri P, Girelli L, Petti M (2023) Network-based validation of the psychometric questionnaire EDI-3 for the assessment of eating disorders. Sci Rep 13:1578. 10.1038/s41598-023-28743-5 36709357 10.1038/s41598-023-28743-5 PMC9884211

[26] Mijas M, Koziara K (2020) Polish adaptation of the daily heterosexist experiences questionnaire. Psychiatr Pol 54:137–152. 10.12740/PP/97215 32447362 10.12740/PP/97215

[27] Harder DW, Rockart L, Cutler L (1993) Additional validity evidence for the harder personal feelings questionnaire-2 (PFQ2): a measure of shame and guilt proneness. J Clin Psychol 49:345–348 8315036 10.1002/1097-4679(199305)49:3<345::aid-jclp2270490307>3.0.co;2-y

[28] Di Sarno M, Di Pierro R, Madeddu F (2022) Shame- and guilt-proneness in an italian sample: latent structure and gender invariance of the personal feelings questionnaire-2 (PFQ-2). Curr Psychol 41:276–288. 10.1007/s12144-019-00570-w

[29] Scheer JR, Antebi-Gruszka N (2019) A psychosocial risk model of potentially traumatic events and sexual risk behavior among LGBTQ individuals. J Trauma Dissociation 20:603–618. 10.1080/15299732.2019.1597815 30932780 10.1080/15299732.2019.1597815 PMC7009774

[30] Harder D, Zalma A (1990) Two promising shame and guilt scales: a construct validity comparison. J Pers Assess 55:729–745. 10.1080/00223891.1990.9674108 2280336 10.1080/00223891.1990.9674108

[31] R Core Team (2024) R: a language and environment for statistical computing. R Foundation for Statistical Computing, Vienna, Austria

[32] Hayes AF (2022) Introduction to mediation, moderation, and conditional process analysis: a regression-based approach, 3rd edn. The Guilford Press, New York, London

[33] James G, Witten D, Hastie T, Tibshirani R (2021) An Introduction to Statistical Learning: with Applications in R. Springer, US, New York, NY

[34] Davidson R, MacKinnon JG (1993) Estimation and inference in econometrics. Oxford University Press, New York Oxford

[35] Hayes AF, Cai L (2007) Using heteroskedasticity-consistent standard error estimators in OLS regression: an introduction and software implementation. Behav Res Methods 39:709–722. 10.3758/BF03192961 18183883 10.3758/bf03192961

[36] McDonald RP (2013) Test Theory: A Unified Treatment. Psychology Press

[37] Hayes AF, Coutts JJ (2020) Use omega rather than Cronbach’s alpha for estimating reliability. But Commun Methods Meas 14:1–24. 10.1080/19312458.2020.1718629

[38] ILGA Europe (2023) What the data says about the acceptance of LGBTI people in Europe. https://www.ilga-europe.org/blog/data-acceptance-lgbti-people-europe/. (Accessed 15 Aug 2025)

[39] Minkin R, Menasce Horowitz J, Braga D (2025) The experiences of LGBTQ Americans Today. In: pew research center. https://www.pewresearch.org/social-trends/2025/05/29/the-experiences-of-lgbtq-americans-today/. (Accessed 15 Aug 2025)

[40] European Union Agency for Fundamental Rights (2024) Harassment and violence against LGBTIQ people on the rise. https://fra.europa.eu/en/news/2024/harassment-and-violence-against-lgbtiq-people-rise. (Accessed 15 Aug 2025)

[41] Guillot L, Coi G (2025) Fundamental rights of LGBTQ+ eroding as they’re weaponized by conservative forces. In: POLITICO. https://www.politico.eu/article/fundamental-rights-lgbtq-eroding-weaponized-conservative-forces-far-right-hate-speech/. (Accessed 30 July 2025)

[42] Van Hoeken D, Hoek HW (2020) Review of the burden of eating disorders: mortality, disability, costs, quality of life, and family burden. Curr Opin Psychiatry 33:521–527. 10.1097/YCO.0000000000000641 32796186 10.1097/YCO.0000000000000641 PMC7575017

[43] Colmsee I-SO, Hank P, Bošnjak M (2021) Low self-esteem as a risk factor for eating disorders: a meta-analysis. Z Psychol 229:48–69. 10.1027/2151-2604/a000433

[44] Leppanen J, Brown D, McLinden H et al (2022) The role of emotion regulation in eating disorders: a network meta-analysis approach. Front Psychiatry 13:793094. 10.3389/fpsyt.2022.793094 35280172 10.3389/fpsyt.2022.793094 PMC8904925

[45] Nechita D, Bud S, David D (2021) Shame and eating disorders symptoms: a meta-analysis. Int J Eat Disord 54:1899–1945. 10.1002/eat.23583 34302369 10.1002/eat.23583

[46] Brewster ME, Velez BL, Esposito J et al (2014) Moving beyond the binary with disordered eating research: a test and extension of objectification theory with bisexual women. J Couns Psychol 61:50–62. 10.1037/a0034748 24188653 10.1037/a0034748

[47] Mezzalira S, Scandurra C, Mezza F et al (2022) Gender felt pressure, affective domains, and mental health outcomes among transgender and gender diverse (TGD) children and adolescents: a systematic review with developmental and clinical implications. Int J Environ Res Public Health 20:785. 10.3390/ijerph20010785 36613106 10.3390/ijerph20010785 PMC9819455

[48] Trombetta T, Paradiso MN, Venturini L et al (2024) The role of adult attachment and minority stress in isolating behaviors perpetration among lesbian and gay people in Italy. Curr Psychol 43:16604–16612. 10.1007/s12144-024-05622-4

[49] Brown TA, Klimek‐Johnson P, Siegel JA et al (2024) Promoting resilience to improve disordered eating (PRIDE): a case series of an eating disorder treatment for sexual minority individuals. Int J Eat Disord 57:648–660. 10.1002/eat.24150 38279188 10.1002/eat.24150

[50] Paulhus DL (1991) Measurement and control of response Bias. In: measures of personality and social psychological attitudes. Elsevier, pp 17–59

[51] Allison DB, Heshka S (1993) Social desirability and response bias in self-reports of “emotional eating.” Eat Disord 1:31–38. 10.1080/10640269308248264

[52] Berinsky AJ, Frydman A, Margolis MF et al (2024) Measuring attentiveness in self-administered surveys. Public Opin Q 88:214–241. 10.1093/poq/nfae004

[53] Krul AJ, Daanen HAM, Choi H (2011) Self-reported and measured weight, height and body mass index (BMI) in Italy, the Netherlands and North America. Eur J Public Health 21:414–419. 10.1093/eurpub/ckp228 20089678 10.1093/eurpub/ckp228

[54] Uniacke B, Glasofer D, Devlin M et al (2021) Predictors of eating-related psychopathology in transgender and gender nonbinary individuals. Eat Behav 42:101527. 10.1016/j.eatbeh.2021.101527 34049054 10.1016/j.eatbeh.2021.101527 PMC8380626

[55] Crenshaw K (1991) Mapping the margins: intersectionality, identity politics, and violence against women of color. Stanf Law Rev 43:1241. 10.2307/1229039

[56] Burke NL, Hazzard VM, Schaefer LM et al (2023) Socioeconomic status and eating disorder prevalence: at the intersections of gender identity, sexual orientation, and race/ethnicity. Psychol Med 53:4255–4265. 10.1017/S0033291722001015 35574702 10.1017/S0033291722001015 PMC9666565

